# Acne tarda: Empfehlungen zu Einordnung, Therapie und Pflege als Ergebnis einer Expertendiskussion

**DOI:** 10.1111/ddg.15913_g

**Published:** 2026-01-14

**Authors:** Matthias Augustin, Thomas Dirschka, Peter Arne Gerber, Martina Kerscher, Natalia Kirsten, Falk Ochsendorf

**Affiliations:** ^1^ Institut für Versorgungsforschung in der Dermatologie und bei Pflegeberufen (IVDP) Universitätsklinikum Hamburg‐Eppendorf (UKE) Hamburg Deutschland; ^2^ CentroDerm Klinik Fakultät für Gesundheit Universität Witten/Herdecke, Witten, Deutschland; ^3^ Dermatologie am Luegplatz Düsseldorf Deutschland Abteilung für Dermatologie Medizinische Fakultät Heinrich‐Heine‐Universität Düsseldorf, Düsseldorf; ^4^ Arbeitsbereich Kosmetikwissenschaften Fachbereich Chemie Universität Hamburg, Hamburg, Deutschland; ^5^ Klinik für Dermatologie Venerologie und Allergologie Universitätsklinikum Frankfurt, Frankfurt am Main, Deutschland

**Keywords:** Acne tarda, Acne vulgaris, Akne, Empfehlungen, Erwachsenenakne, Konsens, Retinoide, Spätakne, Therapie, Acne, acne tarda, acne vulgaris, adult acne, consensus, late onset acne, recommendations, retinoids, treatment

## Abstract

Acne tarda wird in der Literatur als Erwachsenenakne definiert, die laut den meisten Autoren bei Frauen ab 25 Jahren auftritt. Die Definitionen und Altersgrenzen variieren jedoch zwischen den Studien. Gängige Leitlinien berücksichtigen Erwachsenenakne bislang nur selten. In dieser Übersichtsarbeit werden aktuelle Studien und Literatur zur Acne tarda von deutschen Experten zusammengefasst und bewertet. Empfehlungen bezüglich Einordnung, klinischen Merkmalen, Abgrenzung und Therapie der Acne tarda wurden anhand der Diskussion in einem Konsens zusammengefasst. Die Empfehlungen beinhalten zudem die Behandlung postinflammatorischer Erytheme, Hyperpigmentierungen und Aknenarben sowie die begleitende Hautpflege. Ziel ist es, die Versorgung von Patienten mit Acne tarda zu verbessern.

## EINLEITUNG

Die Acne vulgaris, eine entzündliche Erkrankung der Talgdrüsenfollikel, ist weltweit eine der häufigsten Hauterkrankungen, die in einigen Ländern in den letzten 20 Jahren zudem eine steigende Inzidenz aufwies.^1^ In Deutschland blieb die Prävalenz zwar in den letzten Jahren stabil, jedoch wurde hierzulande im Jahr 2019 eine altersstandardisierte Prävalenzrate von 5% festgestellt, welche weltweit unter den Top 20 lag.^1^ Am häufigsten kommt die Akne bei Heranwachsenden vor. Im Alter von 15–18 Jahren beträgt die Prävalenz bis zu 85%.^2^ Akne bedingt für viele Betroffene hohe psychische und soziale Belastungen, die mit starken Einbußen der Lebensqualität einhergehen und zu Depression und erhöhter Suizidgefahr führen können.^3^, ^4^

Unter der Acne vulgaris werden die Acne comedonica (Komedonen dominieren), leichte bis mittelschwere Acne papulopustulosa (entzündliche Papeln und Pusteln dominieren), schwere Acne papulopustulosa/mittelschwere Acne nodularis (Knoten und Zysten vorhanden) sowie die schwere noduläre Acne und Acne conglobata (abszedierende Knoten) zusammengefasst.^2^, ^5^ Acne tarda wird in der Literatur überwiegend definiert als Akne, die über das 25. Lebensjahr hinaus persistiert oder ab diesem Alter erstmals beziehungsweise erneut auftritt.^2^, ^6^, ^7^

Bisher wurden keine grundlegenden Unterschiede in Ursachen und Verlauf der Acne tarda im Vergleich zur adoleszenten Acne vulgaris festgestellt.^8^, ^9^ Obwohl in einer Zwillingsstudie mit erwachsenen weiblichen Zwillingspaaren ein starker Einfluss genetischer Faktoren angenommen wurde, gibt es weiterhin viele ungeklärte Fragen zur Pathogenese der Acne tarda.^10^

Neben der variablen Definition der Acne tarda und der unzureichend untersuchten Pathogenese unterliegt auch deren spezifische Behandlung keinen konsentierten Therapieempfehlungen. Die letzten nationalen und europäischen Leitlinien zur Behandlung der Acne vulgaris wurden bereits vor 7–13 Jahren veröffentlicht, die Therapieempfehlungen bezogen sich jedoch auf die Akne allgemein.^5^, ^11^, ^12^ Bezüglich der Acne tarda empfiehlt die deutsche Leitlinie lediglich Hormonuntersuchungen.^11^ Weitergehende Punkte, wie die weitere Aufarbeitung von Komorbidität, die psychosoziale Diagnostik und Besonderheiten der topischen Basistherapie, werden selten thematisiert.

Vor diesem Hintergrund haben dermatologische Experten aus Deutschland die aktuelle Literatur zur Acne tarda in einem virtuellen Arbeitstreffen gesichtet, diskutiert und bewertet. Anhand einer ersten schriftlichen Zusammenfassung wurden in einer weiteren virtuellen Konferenz Unklarheiten erörtert und in einem Konsens zusammengefasst oder als offene Fragen formuliert. Die Formulierung der Empfehlungsstärke (starke Empfehlung: „soll [nicht]“, Empfehlung: „sollte [nicht]“, offene Empfehlung: „kann erwogen/verzichtet werden“) wurde von allen Experten bestätigt. Abschließend wurden bestehende Behandlungsempfehlungen für die Acne vulgaris im zweiten Treffen evaluiert und um Expertenerfahrungen für die Acne tarda ergänzt. Diese Empfehlungen sollen dazu dienen, Betroffenen mit Acne tarda einen schnelleren Zugang zu angemessenen Therapien und begleitender Hautpflege zu ermöglichen.

## DEFINITION DER ACNE TARDA

Die Acne tarda, auch als Spätakne, Erwachsenenakne, *adult acne* oder postadoleszente Akne bezeichnet, ist in der Literatur überwiegend definiert als Akne, die nach dem 25. Lebensjahr auftritt.^2^, ^6^, ^7^ Die Definition der Acne tarda über das Alter ist jedoch variabel, da einige Lehrbücher, Beobachtungs‐ und klinische Studien die Acne tarda auch ab einem früheren Alter von 18 bis 20 Jahren definieren (Tabelle S1 im Online‐Supplement). In Übersichtsartikeln wird zumeist die Definition „ab 25 Jahren“ verwendet, oder allgemein von Acne tarda als Unterform der Erwachsenenakne bei Frauen gesprochen, welche am Anfang bis Mitte des dritten Lebensjahrzehnt erstmalig auftritt.^8^ Für klinische Studien wird häufig die Definition des Erwachsenen im juristischen Sinne (≥ 18 Jahre) verwendet und dabei hauptsächlich die Begriffe „Akne bei Erwachsenen“, auch „Acne vulgaris bei Erwachsenen“ verwendet, wobei hier das Alter der eingeschlossenen Patienten am häufigsten variierte. Darüber hinaus wird die Acne tarda in der Literatur häufig ausschließlich als Erkrankung der Frau behandelt (Tabelle S1 im Online‐Supplement). Dies kann an der häufig berichteten höheren Prävalenz der adulten Akne bei Frauen liegen, welche aber laut einer systematischen Analyse der Literatur nicht eindeutig belegt ist.^13^

Einige Studien unterscheiden die Acne tarda in zwei Subtypen, jedoch mit abweichenden Kriterien und unterschiedlicher Nomenklatur: Am häufigsten wird die persistierende beziehungsweise die wiederauftretende Acne tarda von der spät erstmanifestierenden Acne tarda differenziert.^14^, ^15^, ^16^, ^17^ Capitanio et al. unterschieden in ihrer Beobachtungsstudie an 226 weiblichen Patienten mit postadoleszenter Akne die klinischen Merkmale der „komedonischen postadoleszenten Akne“ (85% der Betroffenen) sowie der „papulopustulösen postadoleszenten Akne“ anhand ihres klinischen Erscheinungsbildes.^18^ Dabei war die häufigere, komedonische Form mit einem späten Erstauftreten der Akne assoziiert.^18^ Eine ähnliche Unterscheidung in „nichtentzündliche“ und „entzündliche“ Form wird auch von Jansen und Kollegen in einem Übersichtsartikel verwendet.^6^ Zusammengefasst weichen die Definitionen der Acne tarda, des assoziierten Alters und des Geschlechts der Betroffenen in der Literatur deutlich voneinander ab.

### Expertendiskussion zu „Definition der Acne tarda“

Die Experten haben aus der Literatur sowie eigenen Erfahrungen folgende Feststellungen konsentiert:

- Der Begriff „Acne tarda“ soll als Synonym der Erwachsenenakne/Spätakne/postadoleszenten Akne verwendet werden.

Die Experten sind sich, in Übereinstimmung mit der Definition in Lehrbüchern, einig, dass es sich bei der Acne tarda nicht ausschließlich um eine Erstmanifestation der Akne im Erwachsenenalter handelt, sondern auch die über das 25. Lebensjahr hinaus persistierende und rezidivierende Akne unter diesem Begriff eingeschlossen werden soll. Um sicherzustellen, dass es sich nicht um eine über die Pubertät hinaus persistierende, aber dennoch selbstlimitierte Akne handelt, sollte die Acne tarda weiterhin als postadoleszente Akne mit einem Alter von ≥ 25 Jahren definiert werden.

- Die Acne tarda soll per Definition beide Geschlechter umfassen.

Nach Erfahrungen der Experten kann die Acne tarda sowohl das weibliche als auch das männliche Geschlecht betreffen. Laut einer indischen Studie bestehen bei den Merkmalen der Acne tarda keine klinischen Geschlechterunterschiede.^19^ Tendenziell beobachteten die Autoren bei Frauen häufiger Knoten und Zysten, während sich die Acne tarda beim Mann häufiger ähnlich der klassischen Acne vulgaris äußerte. Bei der Frau soll auch der hormonelle Einfluss berücksichtigt werden, gezielt nach Einnahme oder Absetzen von oralen Kontrazeptiva gefragt werden und gegebenenfalls eine Hormonbestimmung in Betracht gezogen werden, um Endokrinopathien wie polyzystisches Ovarialsyndrom (PCOS) oder adrenogenitales Syndrom (AGS) auszuschließen (siehe „Begleitende/assoziierte Erkrankungen und Differenzialdiagnosen“).

- Die Acne tarda kann neben dem Alter über das klinische Erscheinungsbild definiert werden.

Es gibt einige grundlegende klinische Unterschiede zwischen der adoleszenten Acne vulgaris und der Acne tarda, an denen sich die Diagnose der Acne tarda auch orientieren kann (siehe „Klinische Relevanz und Merkmale der Acne tarda**“**).

## PRÄVALENZ DER ACNE TARDA

**ABBILDUNG 1:**
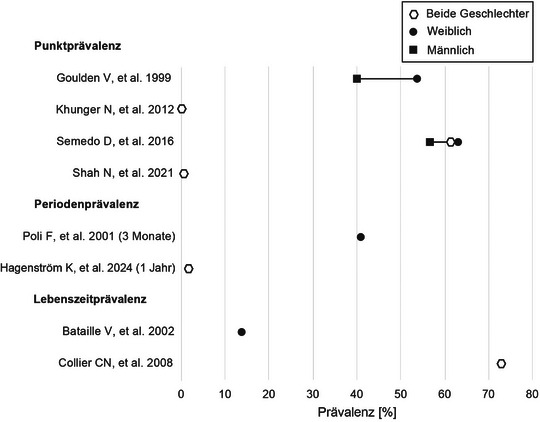
Prävalenzen der in Tabelle 1 genannten Studien mit Fokus auf Acne tarda. Aufgetragen nach Prävalenzart und Geschlecht, wenn angegeben. Fallzahlen wurden nicht berücksichtigt.

Mit Prävalenzen, die je nach Erfassungsstudie zwischen 0,4 und 73,3% variieren, kommt die Acne tarda im Mittel wesentlich häufiger vor als von vielen Dermatologen angenommen (Tabelle 1, Abbildung 1). Die starke Streuung ist vor allem auf unterschiedliche Datenquellen und Erhebungsmethoden, nicht‐standardisierte Studiendesigns, Diagnosekriterien und Beobachtungszeiträume sowie auch Zentrumseffekte zurückzuführen.

**TABELLE 1:** Prävalenz der Acne tarda. Literaturübersicht von Beobachtungsstudien und Selbsteinschätzung mit Fokus auf adulte Akne.

Publikation	Land	Studientyp	Alter [Jahre]	Studien‐population (n)	Prävalenz	Bemerkung
*Punktprävalenz*						
Cunliffe WJ, Gould DJ. 1979^20^	Großbritannien	Umfrage, körperliche Untersuchung	18–70	2133; 1162 ≥ 25	Ca. 30–40% physiologische Akne bei 24 Jahren	Höhere Prävalenz bei Frauen ab 24 Jahren; Abnahme der Prävalenzen mit zunehmendem Alter
Stern RS. 1992^21^	USA	Re‐Evaluation dermatologischer Untersuchung (NHANES‐Studie)	15–44	20 749	27% (Frauen) 34% (Männer)	Höhere Prävalenz bei Männern; auch adoleszente Akne erfasst
Goulden V, et al. 1999^22^	Großbritannien	Zufällige Auswahl und Untersuchung an Klinikum und Umgebung	≥ 25	749	48%	Höhere Prävalenz bei Frauen (54% vs. 40% bei Männern); zu 80% persistierende Akne
Schäfer T, et al. 2001^23^	Deutschland	Standardisiertes Interview und dermatologische Untersuchung	1–87	896 (20–87 Jahre: 761)	26,8% (20–87 Jahre: 26,7%)	Höhere Prävalenz bei Männern (29,9% vs. 23,7% bei Frauen); Korrelation mit Rauchen; auch adoleszente Acne erfasst
Augustin M, et al. 2011^24^	Deutschland	Dermatologische Ganzkörperuntersuchung an Arbeitsstätte	16–70	90 880	3,9%	Anteil Mann/Frau ausgeglichen; auch adoleszente Acne erfasst
Khunger N, et al. 2012^19^	India	Anteil von Aknepatienten in dermatologischer Klinik	≥ 25	72 710	0,38%	Prävalenz berechnet im Verhältnis zu Patienten mit anderen Hauterkrankungen; Höhere Prävalenz bei Frauen; zu 73,2% persistierende Akne
Semedo D, et al. 2016^25^	Portugal	Fragebogen und körperliche Untersuchung	20–60	1055	61,5%	Leichte Akne nur durch klinische Untersuchung festgestellt; kein Unterschied der Prävalenz zwischen Männern und Frauen
Shah N, et al. 2021^26^	Indien	Dermatologische Untersuchung	≥ 25	24 056	0,74%	Unklare Studienpopulation; höhere Prävalenz bei Frauen
Kirsten N, et al. 2021^27^	Deutschland	Dermatologische Ganzkörper‐ Untersuchung in 500 deutschen Firmen	≥ 16	161 269	3,3%	Höchste Prävalenz 16–29 Jahre (9,9%); Abnehmende Prävalenz mit steigendem Alter; auch adoleszente Acne erfasst
*Periodenprävalenz*						
Poli F, et al. 2001^28^	Frankreich	Fragebogen an 4000 erwachsene Frauen; Periodenprävalenz (3 Monate)	25–40	3305	41%	Selbsteinschätzung, nur Frauen eingeschlossen
Thyssen JP, et al. 2022^29^	Dänemark	Nutzung bundesweiter Verwaltungsdaten; Periodenprävalenz (1 Jahr)	12–≥ 40	66 000	3,7%	Kontrollkohorte; Anteil von Frauen mit Akne leicht erhöht; auch adoleszente Acne erfasst
Hagenström K, et al. 2024^30^	Deutschland	Leistungsdatenanalyse (DAK‐Gesundheit); Periodenprävalenz (1 Jahr, für die Jahre 2016–2020 bestimmt)	≥ 25 Jahre und ≥ 30 Jahre	≥ 25: je nach Jahr 1 880 000–2 040 000 ≥ 30: je nach Jahr 1 770 000–1 900 000	≥ 25: 1,5–1,7% ≥ 30: 1,3–1,4% (Raten standardisiert nach Alter und Geschlecht)	Anteil von Frauen erhöht (2020: 73,8% vs. 26,2%)
*Lebenszeitprävalenz*						
Bataille V, et al. 2002^10^	Großbritannien	Weibliche Zwillingsstudie, Fragebogen	18–79	3114	14%	Rückblickende Selbsteinschätzung; nur Frauen, genetischer Zusammenhang festgestellt
Collier CN, et al. 2008^31^	USA	Fragebogen an Universität und Klinikum	≥ 20	1013	73,3%	Selbsteinschätzung jemals Akne gehabt zu haben (auch als Teenager); höhere Prävalenz ≥ 20 Jahren bei Frauen beobachtet

*Abk*.: NHANES, National Health and Nutrition Examination Survey

In der Literatur wird außerdem berichtet, dass ein höherer Anteil männlicher Jugendlicher von der adoleszenten Akne betroffen ist, während anteilig mehr Frauen von Acne tarda berichten.^22^, ^26^, ^31^ Diese Annahme wurde in Prävalenzstudien, die die Acne tarda geschlechtsspezifisch untersuchten, nicht bestätigt (Abbildung 1) und auch in der Literatur kontrovers diskutiert.^13^, ^21^, ^23^

### Expertendiskussion zu „Prävalenz der Acne tarda“

- Zur Bestimmung der genauen Prävalenz der Acne tarda und ihren Unterformen sollten weitere Studien nach standardisierten Einschluss‐ und Diagnosekriterien durchgeführt werden.

Bisherige Studien zur Bestimmung der Prävalenz der Acne tarda sind häufig nicht vergleichbar, da keine konsentierten Einschluss‐ und Diagnosekriterien verwendet wurden. Die Experten würden eine Studie im deutschsprachigen Raum begrüßen, die standardisierte Kriterien verwendet. Hierbei schlagen sie vor, folgende Aspekte zu berücksichtigen:

‐ Eine repräsentative, bevölkerungsbasierte Studienkohorte
‐ Einschluss von Frauen und Männern im Alter ≥ 25 Jahren,
‐ Standardisierte Bewertung der Subtypen von Acne tarda, des Schweregrads im Gesicht und am Rumpf, der Lokalisation und des Ansprechens auf die Behandlung,
‐ Erhebung der subjektiven Belastungen mit validierten patientenberichteten Ergebnissen (*patient‐reported outcomes*, PROs),
‐ Hormonbestimmung bei allen Teilnehmern,
‐ Bestimmung des Therapieansprechens über einen längeren Zeitraum als den gängigen Standard von 12 Wochen.

Eine solche Studie würde einen besseren Überblick über die Situation in Deutschland ermöglichen.

## KLINISCHE RELEVANZ UND MERKMALE DER ACNE TARDA

Die Acne tarda kann die Lebensqualität der älteren Betroffenen stärker beeinträchtigen als die Acne vulgaris bei Jüngeren.^8^, ^32^ Demnach belegte eine auf Skindex‐29‐Fragebögen basierte Studie, dass erwachsene Akne‐Patienten trotz deutlich geringerer Symptomlast ähnliche emotionale und funktionale Beeinträchtigungen erlebten wie Psoriasis‐Patienten.^32^ Zentrale Themen der Akne‐bezogenen emotionalen Auswirkungen sind dabei ein vermindertes Selbstwertgefühl, ein schlechtes Selbstbild, geringes Selbstbewusstsein und Schamgefühle, welche häufig mit einer Abnahme von sozialen Interaktionen einhergehen.^33^ Die Beeinträchtigung der Lebensqualität korrelierte dabei mit dem Schweregrad der Acne tarda, und die psychologischen Auswirkungen wurden immer noch häufig unterschätzt.^8^, ^15^, ^32^ Umso bedeutender ist eine Behandlung mit geeigneten, wirksamen Therapien, denn so kann neben einer Verbesserung des Erscheinungsbildes auch die Lebensqualität der Patienten gesteigert werden.^8^, ^34^, ^35^

Klinisch können laut Literatur zwei Acne tarda‐Typen unterschieden werden:

1. die „nichtentzündliche Acne tarda“ (Acne comedonica tarda), die sich durch große, geschlossene oder zystenartige Komedonen mit einem geringen Anteil entzündlicher Läsionen (< 5%) auszeichnet und
2. die „entzündliche Acne tarda“ (Acne papulopustulosa tarda/ Acne papulo‐pustulo‐nodosa tarda), welche sich durch Papeln, Pusteln und tiefen, entzündlichen Knoten äußert (Anteil entzündlicher Läsionen > 5%), die auch häufig zu Narbenbildung führen.^6^, ^36^

**ABBILDUNG 2:**
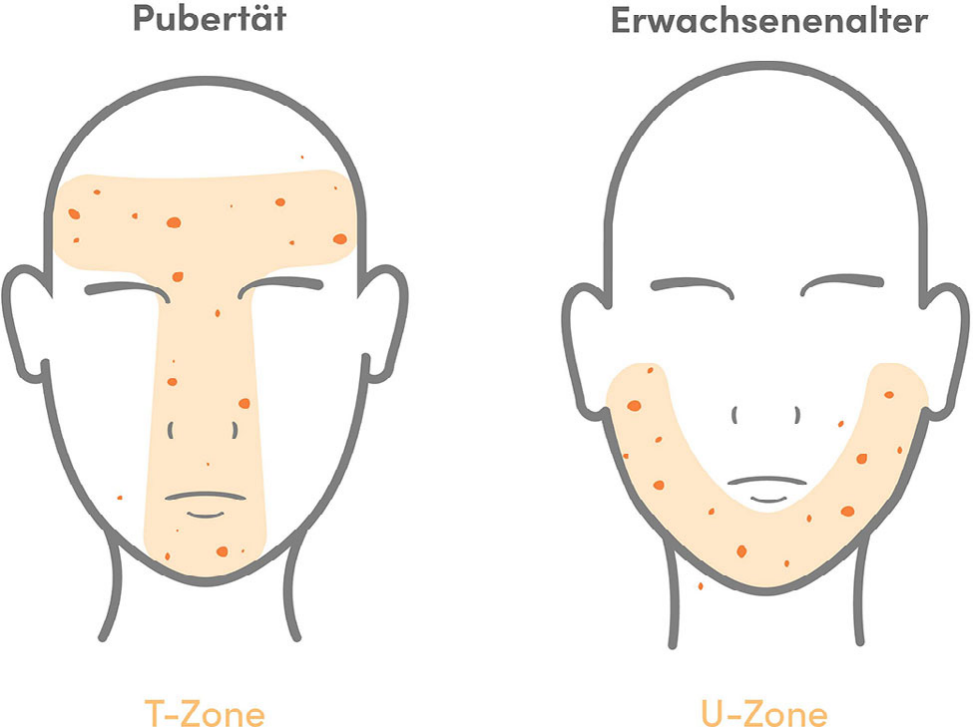
Unterschiede der Akne‐Lokalisation bei Jugendlichen (T‐Zone: Stirn, Nase, oberer Kinnbereich) und Erwachsenen (U‐Zone: Wangen, perioral, unterer Kinnbereich) (adaptiert nach Dréno et al.^8^).

Tatsächlich wurden unterschiedliche Lokalisation der Akne‐Effloreszenzen in Abhängigkeit vom Alter festgestellt. Während bei der adoleszenten Acne vulgaris Papeln, Pusteln und Komedonen vermehrt in der T‐Zone des Gesichts (Stirn, Nase, oberer Kinnbereich) und am Stamm auftreten, ist bei Erwachsenen eher die U‐Zone (Wangen, perioral, unterer Kinnbereich) betroffen (Abbildung 2).^8^, ^37^ Diese Unterschiede könnten auch auf einen jeweils eigenen Pathomechanismus hindeuten.^8^ Darüber hinaus wurde bei der Acne tarda im Gegensatz zur Acne vulgaris häufig die Notwendigkeit einer Langzeittherapie sowie eine erhöhte Narbenbildung beobachtet.^37^, ^38^, ^39^

Zusammengefasst unterscheiden sich die adoleszente Acne vulgaris und die Acne tarda in einigen klinischen Charakteristika. Aufgrund der variablen Definition der Acne tarda ist eine eindeutige Zuordnung jedoch erschwert.

### Expertendiskussion zu „Klinische Relevanz und Merkmale der Acne tarda“

Anhand der Literatur wurden die klinischen Merkmale der Acne vulgaris und der Acne tarda‐Subtypen in Tabelle 2 zusammengefasst und um eigene Erfahrungen der Experten ergänzt.

- Es soll zwischen postinflammatorischen Erythemen (PIE)/postinflammatorischer Hyperpigmentierung (PIH) und Aknenarben unterschieden werden.

**TABELLE 2:** Vergleich der adoleszenten Acne vulgaris und der Acne vulgaris tarda. Bei der Acne tarda wurden auch Merkmale der weiblichen Acne tarda mit einbezogen.

Adoleszente Acne vulgaris	Acne tarda (postadoleszente Akne)	
	*Nichtentzündliche Acne tarda/ Acne comedonica tarda*	*Entzündliche Acne tarda/ Acne papulopustulosa tarda/ Acne papulo‐pustulo‐nodosa tarda*
Am häufigsten in der Pubertät (zwischen 15–18 Jahren)	≥ 25 Jahre	
Komedonen, Papeln, Pusteln^37^	Komedonen, entzündliche Läsionen weniger als 5%^38^	Keine Komedonen, entzündliche Läsionen > 5 %, Papeln, Pusteln und Knoten^6^, ^36^, ^38^
	Eher aus dem Jugendalter persistierend/rezidivierend (Experteneinschätzung); Mit ca. 60–80% die häufigere Form der Acne tarda^15^, ^18^, ^22^, ^28^	Auch Erstmanifestation im Erwachsenenalter (Experteneinschätzung)
Schnelles Auftreten in schwerwiegender Form^37^	Graduelles Auftreten mit hauptsächlich milder/moderater Ausprägung^6^, ^14^, ^37^	
Vermehrt in T‐Zone des Gesichts^8^, ^14^, ^37^	Verteilt, bevorzugt Wangen, Stirn^6^	Vermehrt in U‐Zone des Gesichts (Wangen, perioral, unterer Kinnbereich)^6^, ^37^
Narbenbildung im Vergleich geringer, abhängig des Schweregrades^39^	Narbenbildung nur bei persistierender Form^18^	Erhöhte Narbenbildung, Boxcar‐Narben oder *rolling scars* (tief eingezogene Narben)^18^
Gutes Ansprechen auf Therapie^38^	Besseres Ansprechen auf Therapie als Acne papulopustulosa tarda/ Acne papulo‐pustulo‐nodosa tarda (Experteneinschätzung)	Langzeittherapie notwendig, häufige Rückfälle^6^, ^9^, ^37^, ^38^, ^39^
Vergleichbare Sebum‐Exkretionsraten und *Cutibacterium‐acnes*‐Besiedelung^14^		

Es wird generell berichtet, dass es bei der Acne tarda zu einer erhöhten Narbenbildung kommen kann (Tabelle 2). Es besteht die Annahme, dass diese mit dem erhöhten Anteil entzündlicher Läsionen und der persistierenden Akne in direktem Zusammenhang steht.^39^ Die häufigsten Aknenarben stellen atrophe, eingesunkene Narben dar.^40^ Bei atrophen Narben kommt es im Zuge des Entzündungsprozesses zur vermehrten Expression von Kollagen‐/Elastin‐abbauenden Enzymen (Matrixmetalloproteinasen, MMP; Elastase), was zu einer Degradation von dermalem Gewebe führt. Insgesamt entsteht ein Ungleichgewicht von MMP und deren Regulatoren, den *tissue inhibitors of metalloproteinases* (TIMP), sodass es zu einem Nettoverlust von Gewebe kommt.^41^

Darüber hinaus können, auch durch Selbstschädigung der Haut (Dermatitis factitia), postinflammatorische Erytheme (PIE) oder postinflammatorische Hyperpigmentierungen (PIH) entstehen.^42^ Hierbei sollten PIE/ PIH von Aknenarben unterschieden werden: PIE wird durch die Vasodilatation der oberflächlichen Hautgefäße verursacht, während PIH auf einer durch die Inflammation angeregten Melanogenese beruht, bei der das freigesetzte Melanin in Form von Melanosomen entweder direkt in den Keratinozyten der Epidermis oder über die Aufnahme durch Makrophagen in der Dermis eingelagert wird.^43^ Unbehandelt kann die PIE, mehrere Monate andauern, während die PIH – abhängig von Ursache, Schweregrad der Entzündung und Hauttyp – über Monate bis Jahre oder sogar Jahrzehnte bestehen bleiben kann.^44^ Bei PIE/PIH handelt es sich im Gegensatz zu Aknenarben nicht um Gewebsdefekte, weshalb unterschiedliche Therapieansätze zur erfolgreichen Behandlung vonnöten sind (siehe „Therapiestandards in der Praxis“).

## ÄTIOPATHOGENESE

Es wurde keine deutliche Abweichung der Pathogenese der Acne tarda von der adoleszenten Acne vulgaris festgestellt.^8^, ^9^ Allerdings könnte der Stellenwert der einzelnen pathogenetischen Faktoren bei der Acne tarda unterschiedlich zu bewerten sein.^6^ Grundsätzlich liegen der Entstehung der Akne eine Kombination aus vier pathogenetischen Faktoren zugrunde: *(1)* erhöhte Talgdrüsenaktivität mit vermehrter Sebumproduktion (Seborrhö); *(2)* abnormale Follikeldifferenzierung mit erhöhter Keratinisierung (Verhornungsstörung); *(3)* mikrobielle Überbesiedlung des Follikelkanals mit *Cutibacterium acnes*; *(4)* intra‐ und perifollikuläre Entzündung.^17^, ^45^ Die Inflammation ist ein zentraler pathogenetischer Faktor, denn Akneläsionen können auch ohne klinisch sichtbare Komedonen entstehen und Entzündung kann histologisch bereits vor der sichtbaren Entwicklung einer Akneläsion nachgewiesen werden.^46^, ^47^ Bei Betroffenen mit Acne tarda wurde, vergleichbar zur Acne vulgaris, eine Seborrhö sowie eine gesteigerte Androgen‐Ausschüttung festgestellt. Darüber hinaus werden weitere entzündungsrelevante Signalwege, genetische Prädisposition und externe Auslöser (wie zum Beispiel Ernährung, Stress, Medikation, Rauchen) als zugrundeliegende Faktoren diskutiert.^6^, ^8^, ^9^, ^45^ So wurde bei 37% der Patientinnen mit Acne tarda (definiert ≥ 25 Jahre) Symptome der Hyperandrogenämie festgestellt, während in einer weiteren Studie das Rauchen besonders dem komedonischen Acne tarda‐Typ zugeordnet wurde.^6^, ^18^

Die Sebumproduktion wird nachweislich durch die Ausschüttung von IGF‐1 (*Insulin‐like growth factor 1*, Insulinähnlicher Wachstumsfaktor 1) und Androgenen (Testosteron, Dihydroepiandrosteron‐Sulfat [DHEA‐S]) stimuliert.^17^ So wurde zum Beispiel eine positive Korrelation eines erhöhten IGF‐1‐ und DHEA‐S‐Spiegels bei erwachsenen Frauen mit Acne tarda festgestellt, jedoch nicht bei erwachsenen Männern.^48^ Allerdings fand sich in weiteren Studien kein Zusammenhang zwischen androgenen Hormon‐Serumkonzentrationen und Akne.^37^, ^49^ Da Patientinnen mit Akne häufig vom Auftreten oder Verschwinden der Akneläsionen im Zusammenhang mit Menstruation und Schwangerschaft berichten, herrscht Einigkeit darüber, dass ein hormoneller Einfluss auf die Akne besteht.^6^, ^8^, ^37^ In welchem Ausmaß dies jedoch im Einzelfall relevant ist, bleibt unklar.

Eine genetische Prädisposition zur Entwicklung einer Acne tarda ist wahrscheinlich, da über die Hälfte der Betroffenen von Verwandtschaft ersten Grades mit Acne tarda berichten, beziehungsweise das Risiko einer Acne tarda bei Vorliegen eines entsprechenden verwandtschaftlichen Zusammenhangs erhöht ist.^50^ In einer Zwillingsstudie mit über 1500 erwachsenen weiblichen Zwillingspaaren (458 monozygotisch, 1099 dizygotisch) berichteten von Akne betroffene Zwillinge ebenfalls signifikant häufiger von einer positiven Familienanamnese ersten Grades.^10^ Außerdem wurde durch genetische Modellierung 81% der Akne‐spezifischen Populationsvarianz auf genetische Effekte zurückgeführt. Die verbleibenden 19% wurden individuellen Umweltfaktoren zugeordnet.^10^

Während keine genetischen Untersuchungen spezifisch für die Acne tarda publiziert wurden, konnten mithilfe von genomweiten Sequenziertechniken (*next generation sequencing*, NGS) Acne vulgaris‐assoziierte Suszeptibilitätsloci in Genen des TGF‐β (*transforming growth factor‐β*)‐Signalwegs identifiziert werden (*OVOL1*, *FST*, *TGFB2*).^51^ Eine Abnahme der *OVOL1*‐ und *TGFB2*‐Expression in entzündlichen Akne‐Papeln im Vergleich zur gesunden Haut der gleichen Patienten wurde außerdem bestätigt.^51^ In einer zweiten und dritten Folgestudie wurden insgesamt 43 Loci identifiziert, die mit einer gesteigerten Akne‐Anfälligkeit mit Störungen in Entwicklung, Morphologie und Aktivität des Talgdrüsenfollikels assoziiert sind (unter anderem *WNT10A*, *EDAR*, *LGR6*, *TP63*, *MANC2*).^52^, ^53^ Einzig eine Missense‐Mutation von *WNT10A* zeigte eine höhere Effektstärke bei Männern als bei Frauen, alle weiteren identifizierten Gene zeigten keine geschlechtsspezifischen Unterschiede.^52^ Interessanterweise werden die assoziierten Gene zum Teil mit ektodermalen Dysplasien (*WNT10A*, *TP65*, *EDAR*) und neutrophilen, autoinflammatorischen Dermatosen (generalisierte pustulöse Psoriasis, GPP: *IL36RN*) in Verbindung gebracht, was die Bedeutung der Struktur und Morphologie des Haarfollikels in dermatologischen Erkrankungen hervorhebt.^52^, ^53^ Diese Studien wurden unter Einschluss von Kohorten europäischer Abstammung durchgeführt. Gen‐Assoziationen, die in chinesischen Studien identifiziert wurden, konnten jedoch nicht reproduziert werden, was auf ethnische Unterschiede der zu Akne beitragenden genetischen Faktoren hindeutet.^52^, ^53^ Insgesamt sind damit Prozesse, die zur Entwicklung und Erhaltung des Haarfollikels beitragen, potenzielle neue therapeutische Ansätze neben dem derzeitigen Therapieschema, welches auf die Regulierung von Verhornungsstörung, Entzündung und bakterieller Besiedlung abzielt.

### Expertendiskussion zu „Ätiopathogenese“

Viele grundlegende Vorstellungen zur Entstehung der Akne liegen seit Jahren vor. Wenn man jedoch die hohe Anzahl von Betroffenen und den großen Einfluss der Akne auf deren psychosoziales Wohlbefinden betrachtet, scheint es erstaunlich wenig grundlegende Arbeiten zum Verständnis der exakten Zusammenhänge zur Entstehung der Akne zu geben. Im Folgenden formulierten die Experten weitere, offene Fragen zu Ursachen und Verlauf der Acne tarda:

- Es wird angenommen, dass ca. 400–900 Talgdrüsenfollikel pro cm^2^ im Gesicht existieren. Selbst bei moderater bis schwerer Akne ist nur ein geringer Prozentsatz (0,25%) an sichtbaren Läsionen beteiligt.^54^ Warum?
- Die bis dato durchgeführten Genassoziationsstudien erklären circa 6% der Varianz des Aknerisikos.^53^ Damit sind die familiären Zusammenhänge der Akne zum Großteil weiterhin ungeklärt, und es sind weitere Studien diesbezüglich nötig.
- Welche pathomechanistischen Unterschiede liegen der abweichenden Akne‐Lokalisation bei Jugendlichen (T‐Zone) vs. Erwachsenen (U‐Zone) zugrunde?
- Welche Rolle spielt das Mikrobiom in der Entstehung/Erhaltung der entzündlichen Reaktion?
- Kann durch die Analyse von Biomarkern eine personalisierte Therapie der Akne tarda in der Zukunft ermöglicht werden?
- Wie groß ist der Einfluss von *Lifestyle*‐Faktoren und wie gesichert sind Patienten‐Beratungen dazu?

## BEGLEITENDE/ASSOZIIERTE ERKRANKUNGEN UND DIFFERENZIALDIAGNOSEN

Eine der häufigsten Begleiterkrankungen, die im Zusammenhang mit der Acne tarda auftritt, ist das polyzystische Ovarialsyndrom (PCOS), welches zu circa 30% mit Akne assoziiert ist.^2^ Umgekehrt wurde gezeigt, dass circa 20% der erwachsenen Frauen mit Acne tarda ein PCOS aufwiesen.^49^ Es ist durch Hyperandrogenämie, Insulinresistenz, Oligo‐ und Anovulation sowie polyzystische Ovarien charakterisiert. Akne, Hirsutismus und androgenetische Alopezie sind typische Begleiterscheinungen des PCOS und sollte bei Patientinnen mit Acne tarda auf jeden Fall differenzialdiagnostisch abgeklärt werden.^2^ Weitere Syndrome, die häufig mit Akne assoziiert sind und die mit Hyperandrogenämie, Insulinresistenz oder Autoinflammation einhergehen, sind: Hyperandrogenismus‐Insulinresistenz‐Acanthosis‐nigricans (HAIR‐AN)‐Syndrom, adrenogenitales Syndrom (AGS oder kongenitale adrenale Hyperplasie [CAH]).^2^

Psychosomatische und psychiatrische Komorbidität (Depression, soziale Ängste, körperdysmorphe Störungen, Zwangsstörungen) werden ebenfalls häufig mit der Acne vulgaris in Verbindung gebracht.^55^ Diese beeinträchtigen sowohl adoleszente als auch adulte Patienten mit Akne, die sich häufig von sozialen Aktivitäten wie Ausgehen, Sport und Restaurantbesuchen zurückziehen.^56^ Damit ist die psychische Belastung einer Akne‐Erkrankung nicht zu vernachlässigen und in der Patientenversorgung berücksichtigen.^8^

Insgesamt sollten Begleiterkrankungen erkannt und gegebenenfalls in einem ganzheitlichen Therapieschema berücksichtigt werden. Des Weiteren ist es notwendig, eine Acne tarda von Dermatosen mit ähnlichem klinischem Erscheinungsbild abzugrenzen. In Tabelle 3 sind Differenzialdiagnosen und deren Abgrenzungsmerkmale dargestellt.

**TABELLE 3:** Differenzialdiagnosen der Acne tarda und deren Abgrenzungsmerkmale.

Differenzialdiagnose	Klinische Abgrenzungsmerkmale
Acne excoriée (des jeunes filles)	Polymorphe Läsionen durch beständiges Manipulieren, oft hyperpigmentiert, hämorrhagisch verkrustete Exkoriationen, flache Ulzerationen, die nur langsam abheilen^2^
Acne cosmetica (Kosmetikaakne)	Dicht stehende, überwiegend geschlossene Komedonen und vereinzelte Papulopusteln an Stirn, Wangen, Perioralregion; ausgelöst durch Kosmetika^2^
Mechanische Akne, Teerakne, Chlorakne	Ausgelöst durch Druckstellen/Reibung, Kontakt mit Teer, Vergiftung durch Kohlenwasserstoffverbindungen
Periorale und periorbitale Dermatitis	Hauptsächliche Lokalisation um den Mund beziehungsweise den Augenbereich^8^
Gramnegative Follikulitis	Überwiegend zentrofazial, follikuläre Pusteln auf geröteter Haut, starke Seborrhö^8^, ^17^
Pityrosporum‐Follikulitis	Haarfollikelentzündung mit *Pityrosporum ovale* (zoophiler, lipophiler Hefepilz)^8^, ^17^
Milien	Kleine Keratin enthaltende Zysten; perlweiße Beulen direkt unter der Hautoberfläche; treten meist multipel auf^8^
Rosazea (Pyoderma faciale)	Fehlen von Komedonen, meist keine Seborrhö, Beginn im zentralen Gesichtsbereichs, Augenbeteiligung, Talgdrüsenhyperplasien, oft assoziiert mit Teleangiektasien

### Expertendiskussion zu „Begleitende/assoziierte Erkrankungen und Differenzialdiagnosen“

Neben der Abgrenzung zu den Differenzialdiagnosen empfehlen die Experten folgende Untersuchungen bei der Vorstellung einer Patientin/eines Patienten mit Acne tarda:

- Frauen mit Acne tarda sollten auf Hormonstörungen (PCOS, Hyperandrogenämie) untersucht werden. Dermatologen können die initiale Diagnostik einschließlich der entsprechenden Laboruntersuchungen durchführen. Im Falle eines positiven Befundes für PCOS oder bei Verdacht auf andere endokrinologische Erkrankungen sollte eine Überweisung an einen Endokrinologen/Gynäkologen erfolgen.
- Eine sorgfältige Anamnese und körperliche Untersuchung, die über die Feststellung der Akne hinausgeht, sollte erwogen werden, um klinische Hinweise für eine Hyperandrogenämie zu erkennen (wie Zyklusstörungen, Behaarungsmuster).

## THERAPIESTANDARDS IN DER PRAXIS

Allgemein behalten Therapiestandards und Leitlinien, die für die Aknetherapie der Adoleszenz entwickelt wurden, auch für die Acne tarda ihre Gültigkeit.^5^, ^6^, ^12^, ^57^ Darüber hinaus ist die Therapieadhärenz unabdingbar für den Therapieerfolg.^8^ Die aktuelle deutsche S2k‐Leitlinie „Therapie der Akne“ wurde jedoch vor über 10 Jahren entwickelt, und das letzte Update der europäischen S3‐Leitlinie liegt bereits 7 Jahre zurück, weshalb aktuelle Therapieoptionen der Akne im Therapieschema nicht berücksichtigt werden konnten.^5^, ^12^, ^55^ Auch die Leitlinien des *National Institute for Health and Care Excellence* (NICE) oder die amerikanischen Leitlinien zur Behandlung der Acne vulgaris erwähnen die Acne tarda nicht explizit.^57^, ^58^ Zwar gibt es klinische Studien spezifisch zu der Acne tarda, diese umfassen jedoch zumeist nur kleine Studiengruppen oder es handelt sich um Post‐hoc‐Analysen klinischer Studien zur Acne vulgaris (Tabelle S2 im Online‐Supplement). Selten wird hier die schwere Akne betrachtet und die Acne papulo‐pustulosa‐nodosa tarda generell von den Studien ausgeschlossen (Tabelle S2 im Online‐Supplement).

Ziel der Aknetherapie ist es, die Inflammation schnell und nachhaltig zu reduzieren, Komedonen zu beseitigen beziehungsweise eine Neuentstehung zu verhindern sowie eine Narbenbildung zu vermeiden und mit einer stadiengerechten Therapiewahl die Lebensqualität der Betroffenen zu verbessern. Da hierzu möglichst viele pathogenetische Faktoren gleichzeitig beeinflusst werden sollten, wird eine Kombination mehrerer Substanzen mit additiven oder synergistischen Effekten, präferentiell Fixkombinationen (Adapalen/Benzoylperoxid [BPO], Tretinoin/Clindamycin, Clindamycin/BPO) empfohlen.^2^, ^57^, ^58^

### Expertendiskussion zu „Therapiestandards in der Praxis“

Auf Grundlage der kürzlich aktualisierten, amerikanischen Leitlinien empfehlen die Experten ein ganzheitliches Therapiekonzept, welches um eigene Erfahrungen ergänzt wurde und auch die Hautpflege in Betracht zieht (Tabellen 4, 5).^57^

**TABELLE 4:** Therapieempfehlungen bei der Acne tarda. Die Empfehlungen basieren auf der amerikanischen Leitlinie zur Behandlung der Acne vulgaris bei Erwachsenen, Jugendlichen und Vorpubertierenden (≥ 9 Jahre)^57^ und wurden um Expertenerfahrungen (kursiv) ergänzt.

	Leitlinienempfehlung	Besonderheiten Acne tarda (Expertenerfahrung)
Erste Wahl (deutliche Empfehlung der US‐Leitlinie)	**Leicht**	
	Topische Behandlung (multimodale Kombination empfohlen): ‐ Topische Retinoide (Trifaroten, Adapalen, Tazaroten, Tretinoin, Isotretinoin) ‐ BPO ‐ Topische Antibiotika (keine Monotherapie, nur in Kombination empfohlen)	Topische Retinoide, Azelainsäure und BPO können dauerhaft eingesetzt werden (Erhaltungstherapie)
	Topische Fixkombinationen: ‐ Topische Antibiotika + BPO (z. B. Clindamycin 1% / BPO 5%) ‐ Topische Retinoide + BPO (z. B. Adapalen 0,1% / BPO 2,5%; Adapalen 0,3% / BPO 2,5%) ‐ Topische Retinoide + Antibiotika (z. B. Tretinoin 0,025% / Clindamycin 10%) (gleichzeitige Anwendung von BPO kann Entwicklung von Antibiotikaresistenzen verhindern)	
	**Mittelschwer/schwer** ‐ (Limitierung der antibiotischen Therapie zur Vermeidung einer Resistenzentwicklung; Kombination mit topischer Therapie [außer einer Kombination mit topischen Antibiotika])	
	Systemische Antibiotika^*^: Doxycyclin (bevorzugt gegenüber Azithromycin)	
	Hormonale Behandlung: ‐ Intraläsionale Kortikosteroide (adjuvante Behandlung bei größeren Aknepapeln oder ‐knoten, bei Gefahr von Narbenbildung, oder zur raschen Verbesserung von Entzündungen und Schmerzen)	
	Isotretinoin^**^: ‐ Isotretinoin (bevorzugt täglich; Lidose‐Isotretinoin oder Standard‐Isotretinoin	Isotretinoin‐Indikation großzügiger stellen, um eine Remission einzuleiten Auf Isotretinoin besonders bei Knoten zurückgreifen (Vermeidung von Narbenbildung), bei unzureichender Wirkung auf Knoten intraläsionale Kortikosteroid‐Behandlung 1. Auslassversuch je nach klinischem Ansprechen (ab ca. 6 Monaten)
Zweitlinientherapie (Empfehlung der US‐Leitlinie unter Vorbehalt)	**Leicht**	
	Topische Behandlung: ‐ Clascoteron ‐ Salicylsäure ‐ Azelainsäure	
	**Mittelschwer/schwer**	
	Systemische Antibiotika^*^: ‐ Minocyclin ‐ Sarecyclin	
	Hormonelle Behandlung: ‐ Kombinierte orale Kontrazeptiva ‐ Spironolacton	Hormonale Behandlung nur bei Frauen Spironolacton nach aktueller Studie Doxycyclin überlegen und gut verträglich^59^ Bei Kinderwunsch: Azelainsäure, BPO, Salicylsäure^60^

* Einsatz von Antibiotika limitieren, um das Risiko von Resistenzentwicklung und anderen Antibiotika‐assoziierten Komplikationen zu reduzieren; Kombination mit BPO oder anderen topischen Behandlungen empfohlen.

** Isotretinoin: Patienten mit psychosozialer Belastung oder Narbenbildung sollten als Kandidaten für Isotretinoin in Betracht gezogen werden. Überwachung von Leberfunktion und Lipiden. Populationsbasierte Studien haben kein erhöhtes Risiko für neuropsychiatrische Erkrankungen oder entzündliche Darmerkrankungen unter Isotretinoin festgestellt. Bei Frauen im gebärfähigen Alter ist eine zuverlässige, kontinuierliche Empfängnisverhütung obligatorisch.

*Abk*.: BPO, Benzoylperoxid

**TABELLE 5:** Weitere Maßnahmen bezüglich medizinischer Hautpflege und Behandlung von postinflammatorischen Erythemen (PIE), postinflammatorischen Hyperpigmentierungen (PIH) und Aknenarben.

	*Weitere Maßnahmen bei Acne tarda (Expertenerfahrung)*
**Dermokosmetika**	*Gesichtsreinigung* *Medizinische Kosmetik* *Oberflächliche, chemische Peelings* *Mikrodermabrasion* *Generell: rückfettende Pflege, UV‐Schutz*
**Behandlung von PIE/PIH/ Aknenarben**	***PIE/ PIH***: *Laserbehandlung nicht empfohlen* ***Aknenarben***: *Retinoide (Trifaroten, Adapalen, Tretinoin)* *chemische Peelings* *Dermabrasion* *(Radiofrequenz‐) Microneedling* *Laser (s. S2k‐Leitlinie „Lasertherapie der Haut“)* ^61^

*Abk*.: PIE, postinflammatorische Erytheme; PIH, postinflammatorische Hyperpigmentierung

Demnach sind topische Retinoide bei leichten Formen der Acne tarda (vor allem der Acne comedonica tarda) die Therapie der Wahl, sowohl bei Therapieeinleitung, aber auch als Erhaltungstherapie (Tabelle 4). Dabei werden multimodale Kombinationen empfohlen, wobei auch auf topische Fixkombinationen zurückgegriffen werden kann. Bei mittelschwerer bis schwerer Akne werden zusätzlich systemische Antibiotika der Tetracyclin‐Klasse (vor allem Doxycyclin), sowie eine intraläsionale Kortikosteroidinjektion (zum Beispiel Triamcinolon) zur Therapie von größeren Aknepapeln oder ‐knoten empfohlen. Die Experten befürworten außerdem die Behandlung mit Isotretinoin, besonders bei Knoten beziehungsweise langem Verlauf der Acne tarda. Tetracyclin‐Antibiotika sowie Isotretinoin sind in der Schwangerschaft kontraindiziert, weshalb die Experten eine Empfehlung für Makrolid‐Antibiotika beziehungsweise topisch BPO beziehungsweise Azelainsäure bei Kinderwunsch aussprechen (Tabelle 4).

Die amerikanische Leitlinie enthielt keine Empfehlungen für Licht‐ oder Lasertherapien.^57^ 1726 nm‐Laser, deren Wellenlänge spezifisch auf die Talgdrüse abzielt, stellen künftig potenziell eine Therapieoption für mittelschwere bis schwere Akne dar.^62^, ^63^ Aufgrund der Neuartigkeit der Methode und der limitierten Datenlage, sprechen jedoch weder die amerikanische Leitlinie noch das Expertengremium eine Empfehlung für diese Methode aus.

Weitere Kommentare und Diskussion der Experten zum Therapiealgorithmus:

- Dermokosmetika können bei der Therapie der Acne tarda einen hohen Stellenwert einnehmen.

Die Behandlung mit topischen Retinoiden, Benzoylperoxid (BPO) oder deren Kombination mit weiteren Wirkstoffen sowie mit oralem Isotretinoin führt häufig zu Hautirritationen, Rötungen.^64^ Durch Dosisanpassungen kann versucht werden, diesen unerwünschten Ereignissen vorzubeugen. Dennoch kann auch die Dermokosmetik/rückfettende Pflege einen besonderen Stellenwert einnehmen, um Nebenwirkungen zu reduzieren und insgesamt die Therapieadhärenz zu erhöhen.^65^, ^66^ So zeigte beispielsweise der Einsatz eines nicht fettenden, nicht komedogenen und parfümfreien Produktes eine signifikante Verbesserung von Hauttrockenheit, Rauigkeit, Schuppung sowie Hautunbehagen und Spannungsgefühl nach oraler Isotretinoin‐ oder topischer Tretinointherapie.^64^ Dementsprechend korrelierte in einer Querschnittsstudie die Nutzung begleitender Dermokosmetik mit einer gesteigerten Therapieadhärenz.^67^ Weitere positive Einflussfaktoren auf die Adhärenz waren Akne‐Schweregrad, die klinische Verbesserung, Patientenzufriedenheit und ‐aufklärung.^67^

Dermokosmetische Routinen sollten morgens und abends immer in der Reihenfolge Reinigung, Pflege, Sonnenschutz (nur morgens) erfolgen. Generell werden vor allem sanfte Reinigungsmittel, die den pH‐Wert der Haut aufrechterhalten (salicylhaltige Gels), sowie nicht komedogene Feuchtigkeitscremes, die den Prozess der Barrierereparatur unterstützen, empfohlen.^68^ Da die Integrität der Hautbarriere bei Patienten mit Acne tarda beeinträchtigt ist,^69^ was durch eine Retinoid‐Behandlung noch verstärkt werden kann, ist außerdem ein ausreichender UV‐Schutz (mindestens Lichtschutzfaktor 50) notwendig.^65^ Auch hier sollte auf nichtkomedogene Produkte geachtet werden. Spezialisierte Hautpflegeprodukte mit aktiven Inhaltsstoffen (medizinische Kosmetika) können in Betracht gezogen werden, im Gegensatz zu rein kosmetischen Präparaten, die sich vor allem auf das äußere Erscheinungsbild konzentrieren. Am Abend soll die Gesichtsreinigung wiederholt werden. Auch die Dermokosmetik soll analog zur Acne‐tarda‐Therapie individuell auf die Bedürfnisse der Patienten ausgerichtet werden (Tabelle 5).^65^

- Ein übergreifendes Therapiekonzept beinhaltet auch Vorbeugung und Therapie von postinflammatorischen Erythemen (PIE), postinflammatorischer Hyperpigmentierung (PIH) und Aknenarben.

Bei PIE sollte nach Meinung der Experten keine Laserbehandlung durchgeführt werden. Auch PIH spricht nicht auf die Lasertherapie an und sollte, wenn nötig, mit pharmakologischen Maßnahmen behandelt werden. Aknetherapien können eine Wirkung auf PIH oder Narben haben. Die Behandlung mit Adapalen (0,3%) in Kombination mit BPO (2,5%) führte zu einer reduzierten Bildung atrophischer Narben.^70^ Darüber hinaus wurde eine verringerte Narbenbildung in einer aktuellen, multizentrischen, Vehikel‐kontrollierten Studie zu Trifaroten bestätigt.^71^ Die Ergebnisse zeigten eine signifikante kontinuierliche Reduktion atropher Narben (2–4 mm) von durchschnittlich 11,4 Narben um –5,9 Narben auf der Trifaroten‐Seite vs. –2,7 auf der Vehikel‐Seite nachgewiesen werden.^71^ Demnach können Retinoide zur Vermeidung und Behandlung von Aknenarben empfohlen werden, bevor auf chemische Peelings (zum Beispiel Trichloressigsäure [TCA]‐Peeling), Dermabrasion oder Laser zurückgegriffen werden kann. Die S2k‐Leitlinie „Lasertherapie der Haut“ empfiehlt bei atrophen (Akne‐) Narben zum Beispiel nicht‐ablativ fraktionierte Laser, wie der Er:Glass‐Laser, Farbstoff‐Laser (gepulst) oder IPL (*intense pulsed light*) für erythematöse Aspekte der Narbe, sowie ablative, fraktionierte CO_2_‐ oder Er:YAG‐Laser (Tabelle 5).^61^

- Der Therapieerfolg kann auch über eine messbare Verbesserung der Lebensqualität des Patienten bestimmt werden.

Akne‐spezifische Fragebögen zur Lebensqualität (zum Beispiel Acne Disability Index [ADI], Dermatology Life Quality Index [DLQI]) können laut Experten in Einzelfällen hilfreich sein, um die Lebensqualität (DLQI) oder auch den psychischen Einfluss der Erkrankung (ADI) zu erfassen und so bestimmte Behandlungsentscheidungen zu begründen.

## SCHLUSSFOLGERUNGEN

Diese Übersichtsarbeit und Konsensdiskussion soll dazu dienen, aktuelle Studien und Literatur zur Acne tarda aufzuarbeiten und in Kombination mit eigener Expertise spezifische Empfehlungen bezüglich Einordnung und Behandlung abzuleiten. Die Experten befürworten die Definition der Acne tarda ab einem Alter von 25 Jahren, welche Männer und Frauen betreffen kann. Klinisch wurde die Unterscheidung in zwei Subtypen empfohlen, der Acne comedonica tarda und der Acne papulopustulosa tarda/Acne papulo‐pustulo‐nodosa tarda. Diese unterscheiden sich hauptsächlich in der Anzahl von Komedonen und entzündlichen Läsionen sowie in zu erwartendem Therapieansprechen (Tabelle 2). Bezüglich der Pathogenese der Acne tarda besteht für die Experten der Bedarf an weiteren Studien, um die zahlreichen offenen Fragen zu klären. Begleiterkrankungen sollten erkannt und gegebenenfalls in einem ganzheitlichen Therapieschema berücksichtigt werden. Auf der Basis der bewerteten Studien und der kürzlich aktualisierten amerikanischen/UK Leitlinien, erarbeiteten die Experten Empfehlungen für eine effektive Behandlung der Acne tarda (Tabelle 4). Hierbei wurden auch mögliche Behandlungen von PIE/ PIH und Aknenarben, sowie die Dermakosmetik thematisiert (Tabelle 5). Diese Empfehlungen sollen Dermatologen dabei unterstützen, ihren Patienten einen ganzheitlichen Therapieansatz anbieten zu können.

## DANKSAGUNG

Open access Veröffentlichung ermöglicht und organisiert durch Projekt DEAL.

## INTERESSENKONFLIKT

M.A. hat honorierte Beratungen vorgenommen und/oder Vorträge gehalten und/oder Reisekosten erstattet bekommen und/oder an klinischen Studien teilgenommen die von folgenden Firmen unterstützt wurden: Almirall, Beiersdorf, GSK and Galderma, LEO, l'Oreal, Pierre Fabre,Roche Posay and Viatris. T.D. hat Forschungsgelder von Almirall, Biofrontera, Galderma, Meda und Schulze & Böhm GmbH erhalten; Beratungshonorare von Almirall, Biofrontera, GSK, Dr. Pfleger, Galderma, Janssen‐Cilag, LEO Pharma, Meda, Neracare, Novartis, Scibase, smartinmedia AG, UCB und Vichy; sowie Vortragshonorare von Almirall, Biofrontera, Galderma, GSK, Infectopharm, LEO Pharma, Meda, Neracare, Novartis, Janssen‐Cilag und Riemser. P.A.G. ist Berater und Prüfarzt für Allergan, Galderma und Merz Aesthetics und hat Honorare als Sprecher für Allergan, Croma, Galderma und Merz Aesthetics erhalten. M.K. meldet keine Interessenkonflikte. N.K. war als Referentin und/oder Beraterin für AbbVie, Eli Lilly, Janssen, Novartis, LEO Pharma, UCB und Uluru Inc. tätig und hat an klinischen Studien für AbbVie, Almirall, Boehringer Ingelheim, Eli Lilly, Janssen, Novartis, LEO Pharma, Pfizer, Sanofi und UCB teilgenommen. F.O. war als Berater tätig und hat Vortragshonorare von Galderma, GSK, Mylan und MSD erhalten.

## Supporting information



Supplementary information

Supplementary information
