# Supplementary material for: Acne tarda: Empfehlungen zu Einordnung, Therapie und Pflege als Ergebnis einer Expertendiskussion
Source: J Dtsch Dermatol Ges. 2026 Jan 14;24(1):11–23. [Article in German] doi: 10.1111/ddg.15913_g (PMC12800892; doi:10.1111/ddg.15913_g)
Supplement: Supplementary file 1 — Supplementary information [file DDG-24-11-s001.docx]

**Ergänzende Online-Tabelle 1: Literaturübersicht zur Altersdefinition der Acne tarda.** Publikationen wurden über PubMed mit der Suchanfrage [„acne tarda“ OR „adult acne“] identifiziert.

| Literatur (Auswahl) | Nomenklatur | Alter |
| --- | --- | --- |
| Lehrbücher |  |  |
| Orfanos CE, Garbe C. Therapie der Hautkrankheiten. 2002^1^ | Spättypakne (Acne tarda):  Nach dem 20. Lebensjahr auftretend oder nach dem 25. Lebensjahr oder noch länger persistierend. | ≥ 20 Jahre (Erstauftreten), ≥ 25 Jahre (persistierend) |
| Fritsch P, et al. Dermatologie und Venerologie fürs Studium. 2009^2^ | Acne tarda, als Sonderform der Acne vulgaris. Eine bis ins Erwachsenenalter persistierende Acne bei Frauen. | Erwachsenenalter |
| Zouboulis CC, et al. Pathogenesis and Treatment of Acne and Rosacea. 2014^3^ | Erwachsenenakne, persistierend oder spätauftretend | ≥ 25 Jahre |
| Plewig G, et al. Braun-Falco’s Dermatologie. 2018^4^ | Acne tarda, meist Frauen | ≥ 25 Jahre |
| Übersichtsartikel |  |  |
| Preneau S, Dréno B. 2012^5^ | Akne bei erwachsenen Frauen | ≥ 25 Jahre |
| Jansen T, et al. 2013^6^ | Akne im Erwachsenenalter | ≥ 25 Jahre |
| Zeichner JA, et al. 2017^7^ | Akne bei erwachsenen Frauen | Unterscheidung in:  25 – 44 Jahre und  ≥ 45 Jahre |
| Dréno B, et al. 2018^8^ | Akne bei erwachsenen Frauen:   - Persistierende Akne - Rezidivierende Akne - Acne tarda (Erstauftreten Anfang bis Mitte 20) | „nach der Adoleszenz“ |
| Bagatin E, et al. 2019^9^ | Akne bei erwachsenen Frauen:   - kontinuierlich oder intermittierend aus der Jugend persistierende Akne - neu auftretende Akne | ≥ 25 Jahre |
| Dagnelie MA, et al. 2022^10^ | Akne bei Erwachsenen | „Erwachsenenalter“, ab den Zwanzigern |
| Beobachtungsstudien/ Umfragen |  |  |
| Cunliffe WJ, Gould DJ. 1972^11^ | Gesichtsakne in der späten Jugend und bei Erwachsenen | 18 – 70 Jahre |
| Goulden V, et al. 1999^12^ | Gesichtsakne bei Erwachsenen | ≥ 25 Jahre |
| Poli F, et al. 2001^13^ | Akne bei erwachsenen Frauen | 25 – 40 Jahre |
| Collier CN, et al. 2008^14^ | Akne bei Erwachsenen | ≥ 20 Jahre |
| Khunger N, et al. 2012^15^ | Akne vulgaris bei Erwachsenen | ≥ 25 Jahre |
| Dréno B, et al. 2015^16^ | Akne bei erwachsenen Frauen | ≥ 25 Jahre |
| Semedo D, et al. 2016^17^ | Akne bei Erwachsenen | 20 – 60 Jahre |
| Shah N, et al. 2021^18^ | Akne vulgaris bei Erwachsenen | ≥ 25 Jahre |
| Thyssen JP, et al. 2022^19^ | Akne bei Erwachsenen | 18 – 29, 30 – 39, ≥ 40 Jahre |
| Hagenström K, et al. 2024^20^ | Akne tarda | ≥ 25 Jahre und ≥ 30 Jahre |
| Klinische Studien |  |  |
| Gollnick H, et al. 1999^21^ | Acne tarda des Gesichtstyps | 15 – 50 Jahre |
| Dréno B et al. 2009^22^ | Akne bei erwachsenen Frauen | 30 – 40 Jahre |
| Rademaker M, et al. 2014^23^ | Akne vulgaris bei Erwachsenen | 25 – 55 Jahre |
| Zeichner JA et al. 2015^24^ | Akne vulgaris des Gesichtstyps bei erwachsenen Frauen | ≥ 25 Jahre |
| Thielitz A, et al. 2015^25^ | Akne bei erwachsenen Frauen | 18 – 45 Jahre |
| Stein Gold L et al. 2016^26^ | Akne bei erwachsenen Frauen | ≥ 25 Jahre |
| Alexis AF et al. 2016^27^ | Akne vulgaris bei erwachsenen Frauen | ≥18 Jahre |
| Kainz JT, et al. 2016^28^ | Akne bei erwachsenen Frauen | ≥ 20 Jahre |
| Harper JC, et al. 2019^29^ | Akne vulgaris bei erwachsenen Frauen | ≥ 18 Jahre |
| Chottawornsak N et al. 2019^30^ | Akne bei erwachsenen Frauen | ≥ 25 Jahre |
| Chilicka K, et al. 2020^31^ | Akne bei erwachsenen Frauen | 18 – 25 Jahre |
| Cook-Bolden FE, et al. 2020^32^ | Akne vulgaris bei erwachsenen Männern | ≥ 18 Jahre |
| Patiyasikunt M et al. 2020^33^ | Akne bei erwachsenen Frauen | 25 – 45 Jahre |
| Stein Gold LF, et al. 2022^34^ | Akne bei erwachsenen Frauen | Zwei Kohorten:  18 – 24 Jahre und  ≥ 25 Jahre |
| Dixit N, et al. 2022^35^ | Acne tarda | 25 – 45 Jahre |
| Gerber PA. 2023^36^ | Altersakne, Acne tarda | ≥ 25 Jahre |
| Santer M, et al. 2023^37^ | Akne vulgaris bei erwachsenen Frauen | ≥ 18 Jahre |
| Dréno B, et al. 2024^38^ | Akne bei erwachsenen Frauen | ≥ 20 Jahre |

**Referenzen**

1. Orfanos CE, Garbe C. in Therapie der Hautkrankheiten: einschließlich Allergologie, Andrologie, Phlebologie, Proktologie, Trichologie, pädiatrische Dermatologie, tropische Dermatosen, Venerologie und HIV-Infektion sowie dermatologische Notfälle (eds Constantin E. Orfanos *et al.*) 377-417 (Springer Berlin Heidelberg, 2002).

2. Fritsch P. in Dermatologie und Venerologie für das Studium (ed Peter Fritsch) 444-490 (Springer Berlin Heidelberg, 2009).

3. Lucky AW, Dessinioti C, Katsambas AD. in Pathogenesis and Treatment of Acne and Rosacea (eds Christos C. Zouboulis, Andreas D. Katsambas, & Albert M. Kligman) 243-249 (Springer Berlin Heidelberg, 2014).

4. Plewig G, Ruzicka T, Kaufmann R et al. Braun-Falco's Dermatologie, Venerologie und Allergologie. 7., vollständig überarbeitete und aktualisierte Auflage. edn, (Springer, 2018).

5. Preneau S, Dréno B. Female acne - a different subtype of teenager acne? *J Eur Acad Dermatol Venereol.* 2012;26:277-282.

6. Jansen T, Janssen OE, Plewig G. [Acne tarda. Acne in adults]. *Hautarzt.* 2013;64:241-251.

7. Zeichner JA, Baldwin HE, Cook-Bolden FE et al. Emerging Issues in Adult Female Acne. *J Clin Aesthet Dermatol.* 2017;10:37-46.

8. Dréno B, Bagatin E, Blume-Peytavi U et al. Female type of adult acne: Physiological and psychological considerations and management. *J Dtsch Dermatol Ges.* 2018;16:1185-1194.

9. Bagatin E, Freitas THP, Rivitti-Machado MC et al. Adult female acne: a guide to clinical practice. *An Bras Dermatol.* 2019;94:62-75.

10. Dagnelie MA, Poinas A, Dréno B. What is new in adult acne for the last 2 years: focus on acne pathophysiology and treatments. *Int J Dermatol.* 2022;61:1205-1212.

11. Cunliffe WJ, Gould DJ. Prevalence of facial acne vulgaris in late adolescence and in adults. *Br Med J.* 1979;1:1109-1110.

12. Goulden V, Stables GI, Cunliffe WJ. Prevalence of facial acne in adults. *J Am Acad Dermatol.* 1999;41:577-580.

13. Poli F, Dréno B, Verschoore M. An epidemiological study of acne in female adults: results of a survey conducted in France. *J Eur Acad Dermatol Venereol.* 2001;15:541-545.

14. Collier CN, Harper JC, Cafardi JA et al. The prevalence of acne in adults 20 years and older. *J Am Acad Dermatol.* 2008;58:56-59.

15. Khunger N, Kumar C. A clinico-epidemiological study of adult acne: is it different from adolescent acne? *Indian J Dermatol Venereol Leprol.* 2012;78:335-341.

16. Dréno B, Thiboutot D, Layton AM et al. Large-scale international study enhances understanding of an emerging acne population: adult females. *J Eur Acad Dermatol Venereol.* 2015;29:1096-1106.

17. Semedo D, Ladeiro F, Ruivo M et al. Adult Acne: Prevalence and Portrayal in Primary Healthcare Patients, in the Greater Porto Area, Portugal. *Acta Med Port.* 2016;29:507-513.

18. Shah N, Shukla R, Chaudhari P et al. Prevalence of acne vulgaris and its clinico-epidemiological pattern in adult patients: Results of a prospective, observational study. *J Cosmet Dermatol.* 2021;20:3672-3678.

19. Thyssen JP, Nymand LK, Maul JT et al. Incidence, prevalence and risk of acne in adolescent and adult patients with atopic dermatitis: a matched cohort study. *J Eur Acad Dermatol Venereol.* 2022;36:890-896.

20. Hagenström K, Müller K, Klinger T et al. Epidemiology and Healthcare of Juvenile and Late-Onset Acne: Claims Data Analysis. *Dermatol Ther (Heidelb).* 2024;

21. Gollnick H, Albring M, Brill K. [The effectiveness of oral cyproterone acetate in combination with ethinylestradiol in acne tarda of the facial type]. *Ann Endocrinol (Paris).* 1999;60:157-166.

22. Dréno B, Castell A, Tsankov N et al. Interest of the association retinaldehyde/glycolic acid in adult acne. *J Eur Acad Dermatol Venereol.* 2009;23:529-532.

23. Rademaker M, Wishart JM, Birchall NM. Isotretinoin 5 mg daily for low-grade adult acne vulgaris--a placebo-controlled, randomized double-blind study. *J Eur Acad Dermatol Venereol.* 2014;28:747-754.

24. Zeichner JA. The Efficacy and Tolerability of a Fixed Combination Clindamycin (1.2%) and Benzoyl Peroxide (3.75%) Aqueous Gel in Adult Female Patients with Facial Acne Vulgaris. *J Clin Aesthet Dermatol.* 2015;8:21-25.

25. Thielitz A, Lux A, Wiede A et al. A randomized investigator-blind parallel-group study to assess efficacy and safety of azelaic acid 15% gel vs. adapalene 0.1% gel in the treatment and maintenance treatment of female adult acne. *J Eur Acad Dermatol Venereol.* 2015;29:789-796.

26. Gold LS, Baldwin H, Rueda MJ et al. Adapalene-benzoyl Peroxide Gel is Efficacious and Safe in Adult Female Acne, with a Profile Comparable to that Seen in Teen-aged Females. *J Clin Aesthet Dermatol.* 2016;9:23-29.

27. Alexis AF, Burgess C, Callender VD et al. The Efficacy and Safety of Topical Dapsone Gel, 5% for the Treatment of Acne Vulgaris in Adult Females With Skin of Color. *J Drugs Dermatol.* 2016;15:197-204.

28. Kainz JT, Berghammer G, Auer-Grumbach P et al. Azelaic acid 20 % cream: effects on quality of life and disease severity in adult female acne patients. *J Dtsch Dermatol Ges.* 2016;14:1249-1259.

29. Harper JC, Baldwin H, Stein Gold L, Guenin E. Efficacy and Tolerability of a Novel Tretinoin 0.05% Lotion for the Once-Daily Treatment of Moderate or Severe Acne Vulgaris in Adult Females. *J Drugs Dermatol.* 2019;18:1147-1154.

30. Chottawornsak N, Chongpison Y, Asawanonda P, Kumtornrut C. Topical 2% ketoconazole cream monotherapy significantly improves adult female acne: A double-blind, randomized placebo-controlled trial. *J Dermatol.* 2019;46:1184-1189.

31. Chilicka K, Rogowska AM, Szygula R et al. A comparison of the effectiveness of azelaic and pyruvic acid peels in the treatment of female adult acne: a randomized controlled trial. *Sci Rep.* 2020;10:12612.

32. Cook-Bolden FE, Gold MH, Guenin E. Tazarotene 0.045% Lotion for the Once-Daily Treatment of Moderate-to-Severe Acne Vulgaris in Adult Males. *J Drugs Dermatol.* 2020;19:78-85.

33. Patiyasikunt M, Chancheewa B, Asawanonda P et al. Efficacy and tolerability of low-dose spironolactone and topical benzoyl peroxide in adult female acne: A randomized, double-blind, placebo-controlled trial. *J Dermatol.* 2020;47:1411-1416.

34. Stein Gold L, Kircik L, Baldwin H et al. Tazarotene 0.045% Lotion for Females With Acne: Analysis of Two Adult Age Groups. *J Drugs Dermatol.* 2022;21:587-595.

35. Dixit N, Jena A, Panda M et al. Randomized prospective study of low-dose isotretinoin alone and combination with salicylic acid and mandelic peel against acne tarda. *J Cosmet Dermatol.* 2022;21:4398-4404.

36. Gerber PA. Akne – Bewährtes und Neues. *Thieme Kompendium Dermatologie.* 2023:28–34.

37. Santer M, Lawrence M, Renz S et al. Effectiveness of spironolactone for women with acne vulgaris (SAFA) in England and Wales: pragmatic, multicentre, phase 3, double blind, randomised controlled trial. *BMJ.* 2023;381:e074349.

38. Dréno B, Nguyen JM, Hainaut E et al. Efficacy of Spironolactone Compared with Doxycycline in Moderate Acne in Adult Females: Results of the Multicentre, Controlled, Randomized, Double-blind Prospective and Parallel Female Acne Spironolactone vs doxyCycline Efficacy (FASCE) Study. *Acta Derm Venereol.* 2024;104:adv26002.
