# Supplementary material for: Acne tarda: Empfehlungen zu Einordnung, Therapie und Pflege als Ergebnis einer Expertendiskussion
Source: J Dtsch Dermatol Ges. 2026 Jan 14;24(1):11–23. [Article in German] doi: 10.1111/ddg.15913_g (PMC12800892; doi:10.1111/ddg.15913_g)
Supplement: Supplementary file 2 — Supplementary information [file DDG-24-11-s002.docx]

**Ergänzende Online-Tabelle 2: Übersicht zu klinischen Studien zur Therapiewirksamkeit bei Acne tarda.** AFA = Adult female acne; AFAST-F = Adult Female Acne Scoring Tool - Face; EGSS = Evaluator’s Global Severity Score; GAAS = Global Acne Assessment Score; IGA = Investigator's Global Assessment; ISGA = Investigator's Static Global Assessment; PGA = Physician's Global Assessment. Relevante Studien, Stand Februar 2025.

| **Autoren** | **Studientyp, Untersuchungsgruppe** | **Aknetyp** | **Intervention** |
| --- | --- | --- | --- |
| Gollnick H, et al. 1999^1^ | Offene, multizentrische Studie; n=890: Frauen, 15–50 Jahre | Alle Typen: offene und geschlossene Komedonen, Papeln, Pusteln, Knötchen und Zysten | 2 mg Cyproteronacetat + 0,035 mg Ethinyloestradiol |
| Dréno B, et al. 2009^2^ | Offene, multizentrische Studie; n=397: Frauen 30–40 Jahre | Akne, ohne nähere Angaben | Retinaldehyd (0,1 %) + Glykolsäure (6 %) |
| Rademaker M, et al. 2014^3^ | Randomisierte, placebokontrollierte Studie; n=60: Frauen und Männer, 22–55 Jahre | Leichte Akne: ≥3 Akneläsionen/Monat im Gesicht für ≥3 Monate; Grad 1–2 Modified Leeds Acne Assessment scale | Low-dose (5 mg) Isotretinoin vs. Placebo |
| Zeichner JA, et al. 2015^4^ | Post-hoc Subgruppenanalyse; n=72: Frauen, ≥25 Jahre | Mittelschwere bis schwere Acne vulgaris: 20–40 Papeln/ Pusteln/ Knoten, 20–100 offene/ geschlossene Komedonen), ≤2 Knoten | Clindamycin (1,2 %)/ Benzoylperoxid (3,75%) Gel vs. Vehikel |
| Thielitz A, et al. 2015^5^ | Randomisierte, monozentrische, placebokontrollierte Studie; n=55: Frauen, 18–45 Jahre | Leichte bis mittelschwere Akne: Grad 2–4 nach modifiziertem ISGA oder 2–7 nach Leeds Revised Acne Grading Scale; ≤1 Knoten | Azelainsäure (15 %) Gel vs. Adapalen (0,1 %) Gel |
| Stein Gold L, et al. 2016^6^ | Meta-Subgruppenanalyse randomisierter, placebokontrollierter Studien; n=254: Frauen, ≥25 Jahre | Leichte, mittelschwere und schwere Akne nach IGA | Adapalen (0,1 %)/ Benzoylperoxid (2,5 %) Gel vs. Vehikel |
| Alexis AF, et al. 2016^7^ | Offene, multizentrische Studie; n=68: Frauen, ≥18 Jahre, farbige Haut (Fitzpatrick-Typen IV-VI) | Gesichtsakne nach GAAS | Dapson (5 %) Gel |
| Kainz JT, et al. 2016^8^ | Prospektive, nicht-interventionelle Studie; n=251: Frauen ≥20 Jahre | Leichte bis mittelschwere Akne (IGA Grad 1–3) | Azelainsäure (20 %)-Salbe |
| Harper JC, et al. 2019^9^ | Post-hoc Subgruppenanalyse von 2 multizentrischen, randomisierten, placebokontrollierten Studien; n=606; Frauen ≥18 Jahre | Mittelschwere bis schwere Acne vulgaris nach EGSS | Tretinoin (0,05 %) Lotion vs. Vehikel |
| Chottawornsak N, et al. 2019^10^ | Randomisierte, monozentrische, placebokontrollierte Studie; n=41: Frauen, ≥25 Jahre | Leichte Akne (AFA Score 2: ≤50% des Gesichts betroffen, wenige Komedonen, Papeln, Pusteln)^11^ | Ketoconazol (2 %)-Salbe vs. Vehikel |
| Patiyasikunt M, et al. 2020^12^ | Randomisierte, monozentrische, placebokontrollierte Studie; n=60: Frauen, 25–45 Jahre | Mittelschwere Akne Grad 3 gemäß AFAST-F^13^ | Low-dose Spironolacton (25/50 mg) + Benzoylperoxid 2,5 % Gel vs. Vehikel |
| Chilicka K, et al. 2020^14^ | Randomisierte, monozentrische Studie; n=120: Frauen, 18–24 Jahre | Leichte bis mittelschwere papulopustulöse Akne | Azelainsäure (16 %)- vs. Brenztraubensäure (50 %)-Peeling |
| Cook-Bolden FE et al. 2020^15^ | Post-hoc Subgruppenanalyse von 2 multizentrischen, randomisierten, placebokontrollierten Studien; n=268: Männer ≥18 Jahre | Mittelschwere bis schwere Acne vulgaris: 20–50 Papeln/ Pusteln/ Knoten, 25–100 offene/ geschlossene Komedonen), ≤2 Knoten | Tazaroten (0,045 %)-Lotion vs. Vehikel |
| Stein Gold L, et al. 2022^16^ | Post-hoc Subgruppenanalyse von 2 multizentrischen, randomisierten, placebokontrollierten Studien; n=744: Frauen ≥18 Jahre | Mittelschwere bis schwere Acne vulgaris: 20–50 Papeln/ Pusteln/ Knoten, 25–100 offene/ geschlossene Komedonen), ≤2 Knoten | Tazaroten (0,045 %)-Lotion vs. Vehikel |
| Gerber PA. 2023^17^ | Post-hoc Subgruppenanalyse einer multizentrischen Phase-III-Langzeitstudie; n=46: Frauen ≥25 Jahre | Mittelschwere Akne: IGA/PGA=3; ≥20 entzündliche Läsionen und ≥25 nicht-entzündliche Läsionen im Gesicht; ≥20 entzündliche Läsionen und ≥20 nicht entzündliche Läsionen am Rumpf | Trifaroten (50 µg/g Creme) |
| Santer M, et al. 2023^18^ | Multizentrische, randomisiert kontrollierte Phase-III-Studie (UK, Wales), n=410; Frauen ≥18 Jahre | Gesichtsakne seit mindestens 6 Monaten; IGA ≥2 | Spironlacton (50mg pro Tag) oder Placebo, nach 6 Wochen auf 100 mg/Tag Spironolacton oder Placebo erhöht |
| Dréno B, et al. 2024^19^ | Multizentrische, randomisierte, kontrollierte, doppelblinde prospektive und parallele Studie (Frankreich), n=133; Frauen ≥20 Jahre | Mittelschwere Akne (mindestens 10 entzündliche Läsionen und nicht mehr als 3 Knötchen nach AFAST-Score an Gesicht und Unterkiefer) | Spironlacton (150 mg pro Tag) vs. Doxycyclin (100 mg pro Tag); beide + 5 % BPO |
| Baldwin H, et al. 2024^20^ | 2 multizentrische, randomisierte, kontrollierte, doppelblinde Phase-III-Studien mit Patienten ≥9 Jahre (n=363). Post hoc Subgruppenanalyse für adulte Patienten ≥18 Jahre (n=185) | Mittelschwere bis schwere Akne bach EGSS (Grad 3 oder 4); 30–100 entzündliche Läsionen im Gesicht; 35–150 nichtentzündliche Läsionen und ≤2 Gesichts-Knötchen. | Topisches Clindamycinphosphat 1,2 %/ Adapalen 0,15 %/ Benzoylperoxid 3,1 % Gel (CAB; Dreifachkombination) vs. Vehikel-Gel |

**Referenzen**

1. Gollnick H, Albring M, Brill K. [The effectiveness of oral cyproterone acetate in combination with ethinylestradiol in acne tarda of the facial type]. *Ann Endocrinol (Paris).* 1999;60:157-166.

2. Dréno B, Castell A, Tsankov N et al. Interest of the association retinaldehyde/glycolic acid in adult acne. *J Eur Acad Dermatol Venereol.* 2009;23:529-532.

3. Rademaker M, Wishart JM, Birchall NM. Isotretinoin 5 mg daily for low-grade adult acne vulgaris--a placebo-controlled, randomized double-blind study. *J Eur Acad Dermatol Venereol.* 2014;28:747-754.

4. Zeichner JA. The Efficacy and Tolerability of a Fixed Combination Clindamycin (1.2%) and Benzoyl Peroxide (3.75%) Aqueous Gel in Adult Female Patients with Facial Acne Vulgaris. *J Clin Aesthet Dermatol.* 2015;8:21-25.

5. Thielitz A, Lux A, Wiede A et al. A randomized investigator-blind parallel-group study to assess efficacy and safety of azelaic acid 15% gel vs. adapalene 0.1% gel in the treatment and maintenance treatment of female adult acne. *J Eur Acad Dermatol Venereol.* 2015;29:789-796.

6. Gold LS, Baldwin H, Rueda MJ et al. Adapalene-benzoyl Peroxide Gel is Efficacious and Safe in Adult Female Acne, with a Profile Comparable to that Seen in Teen-aged Females. *J Clin Aesthet Dermatol.* 2016;9:23-29.

7. Alexis AF, Burgess C, Callender VD et al. The Efficacy and Safety of Topical Dapsone Gel, 5% for the Treatment of Acne Vulgaris in Adult Females With Skin of Color. *J Drugs Dermatol.* 2016;15:197-204.

8. Kainz JT, Berghammer G, Auer-Grumbach P et al. Azelaic acid 20 % cream: effects on quality of life and disease severity in adult female acne patients. *J Dtsch Dermatol Ges.* 2016;14:1249-1259.

9. Harper JC, Baldwin H, Stein Gold L, Guenin E. Efficacy and Tolerability of a Novel Tretinoin 0.05% Lotion for the Once-Daily Treatment of Moderate or Severe Acne Vulgaris in Adult Females. *J Drugs Dermatol.* 2019;18:1147-1154.

10. Chottawornsak N, Chongpison Y, Asawanonda P, Kumtornrut C. Topical 2% ketoconazole cream monotherapy significantly improves adult female acne: A double-blind, randomized placebo-controlled trial. *J Dermatol.* 2019;46:1184-1189.

11. Dréno B, Poli F, Pawin H et al. Development and evaluation of a Global Acne Severity Scale (GEA Scale) suitable for France and Europe. *J Eur Acad Dermatol Venereol.* 2011;25:43-48.

12. Patiyasikunt M, Chancheewa B, Asawanonda P et al. Efficacy and tolerability of low-dose spironolactone and topical benzoyl peroxide in adult female acne: A randomized, double-blind, placebo-controlled trial. *J Dermatol.* 2020;47:1411-1416.

13. Poli F, Auffret N, Claudel JP et al. AFAST: an adult female acne treatment algorithm for daily clinical practice. *Eur J Dermatol.* 2018;28:101-103.

14. Chilicka K, Rogowska AM, Szygula R et al. A comparison of the effectiveness of azelaic and pyruvic acid peels in the treatment of female adult acne: a randomized controlled trial. *Sci Rep.* 2020;10:12612.

15. Cook-Bolden FE, Gold MH, Guenin E. Tazarotene 0.045% Lotion for the Once-Daily Treatment of Moderate-to-Severe Acne Vulgaris in Adult Males. *J Drugs Dermatol.* 2020;19:78-85.

16. Stein Gold L, Kircik L, Baldwin H et al. Tazarotene 0.045% Lotion for Females With Acne: Analysis of Two Adult Age Groups. *J Drugs Dermatol.* 2022;21:587-595.

17. Gerber PA. Akne – Bewährtes und Neues. *Thieme Kompendium Dermatologie.* 2023:28–34.

18. Santer M, Lawrence M, Renz S et al. Effectiveness of spironolactone for women with acne vulgaris (SAFA) in England and Wales: pragmatic, multicentre, phase 3, double blind, randomised controlled trial. *BMJ.* 2023;381:e074349.

19. Dréno B, Nguyen JM, Hainaut E et al. Efficacy of Spironolactone Compared with Doxycycline in Moderate Acne in Adult Females: Results of the Multicentre, Controlled, Randomized, Double-blind Prospective and Parallel Female Acne Spironolactone vs doxyCycline Efficacy (FASCE) Study. *Acta Derm Venereol.* 2024;104:adv26002.

20. Baldwin H, Gold LS, Harper JC et al. Triple-Combination Clindamycin Phosphate 1.2%/Adapalene 0.15%/Benzoyl Peroxide 3.1% Gel for Acne in Adult and Pediatric Participants. *J Drugs Dermatol.* 2024;23:394-402.
